# Efficacy of flaxseed in reducing blood pressure among patients with cardiovascular risk factors: A systematic review and meta-analysis of parallel randomized controlled trials

**DOI:** 10.34172/jcvtr.025.33280

**Published:** 2025-03-18

**Authors:** Refli Hasan, Raed Obaid Saleh, Rana H. Raheema, Hanen Mahmod Hulail, Irfan Ahmad, Deepak Nathiya, Parjinder Kaur

**Affiliations:** ^1^Department of Internal Medicine, Faculty of Medicine, Universitas Sumatera Utara, Medan, Indonesia; ^2^Medical Laboratory Techniques Department, College of Health and Medical Technology, University of Al Maarif, Anbar, Iraq; ^3^Department of Medical Microbiology, College of Medicine, Wasit University, Kut, Iraq; ^4^Department of Medical Laboratories Technology, AL-Nisour University College, Baghdad, Iraq; ^5^Central Labs, King Khalid University, AlQura’a, Abha, Saudi Arabia; ^6^Department of Clinical Laboratory Sciences, College of Applied Medical Sciences, King Khalid University, Abha, Saudi Arabia; ^7^Department of Pharmacy Practice, NIMS Institute of Pharmacy, NIMS University Rajasthan, Jaipur, India; ^8^Chandigarh Pharmacy College, Chandigarh Group of Colleges-Jhanjeri, Mohali 140307, Punjab, India

**Keywords:** Flaxseed, Blood pressure, Cardiovascular disease, Systematic review, Meta-analysis

## Abstract

The prevalence rate of hypertension is on the rise at an alarming rate. Studies conducted on the influence of flaxseed on blood pressure (BP) have come up with conflicting conclusions. The current investigation’s major purpose is to conduct a literature review and a meta-analysis focusing on the effect of flaxseed supplementation on BP in people with cardiovascular disease (CVD) risk factors. PubMed, Scopus, Web of Science, and Cochrane Central Library databases were searched from the inception date to April 2024 to find the randomized controlled trials (RCTs). A random-effects model combined the weighted mean difference (WMD). Standard methodologies were applied to evaluate publication bias, heterogeneity, and sensitivity analysis. Eighteen RCTs were included in the present systematic review and meta-analysis. Pooled analysis suggested that flaxseed supplementation can reduce systolic BP (SBP) (WMD: -4.75 mmHg, 95% CI: -7.05 to -2.44, *P*≤0.001; I^2^=93.6%) and diastolic BP (DBP) (WMD: -3.09 mmHg, 95% CI: -4.37 to -1.81, *P*≤0.001; I^2^=91.2%). In conclusion, the current meta-analysis has demonstrated that flaxseed supplementation can markedly lower BP in individuals exhibiting CVD risk factors. Given the significant heterogeneity, it is crucial to interpret the current results with careful consideration. In addition, further high-quality RCTs are required to better assess the causal relationships.

## Introduction

 Cardiovascular disease (CVD) is the foremost cause of mortality across the globe, making it a critical public health concern. Significant cardiovascular risk determinants encompass factors such as obesity, age, sex, dyslipidemia, metabolic syndrome (MetS), and type 2 diabetes mellitus (T2DM).^1,2^ Another significant risk factor for cardiovascular and renal diseases is hypertension (HTN). It is estimated that 1.3 billion adults worldwide have HTN, and it is mostly prevalent in low- and middle-income countries. HTN is present in about 25% of the adult population and only one-third of these patients manage to control their blood pressure (BP).^3^ Therefore, controlling BP is crucial for any healthcare system as HTN incurs significant costs for the government and a burden on the community.^3,4^ According to experts, a combination of lifestyle changes, such as increased consumption of fruits, low-fat dairy, vegetables, sodium reduction, and exercise together with medication is critical in the management of BP.^5-7^ Due to the various adverse effects of pharmaceutical therapy, nutrition therapy has gained increasing attention in recent years.^8-10^ In this sense, herbal treatments become important due to their efficacy in treating high BP and their low cost.^11-14^ It’s important to note that herbal compounds should be used as a complementary solution alongside aforesaid approaches to control BP.^15-17^

 Flaxseed also known as linseed (*Linum usitatissimum*) is a functional food rich in alpha-linolenic acid (ALA) and effective phytochemicals including lignan, phenolic acids, phytoestrogens, and flavonoids.^18,19^ It possesses several properties such as antioxidant,^20^ anti-atherosclerotic,^18,21-23^ anti-obesity,^24^ anti-diabetic,^25^ anti-microbial,^26^ anti-cancer ^27^, anti-arthritic^28^ and anti-inflammatory.^21^ Given the aforementioned properties and the high content of polyunsaturated fatty acids (PUFA) in flaxseed, it is anticipated that flaxseed may also be effective in improving BP. A multitude of trials has been conducted to assess the efficacy of flaxseed and its derivatives in regulating BP among human subjects.^29-46^ However, the results of the existing clinical trials have been inconsistent, and the differences might be due to various aspects of the study design, such as study power, the recruitment of patients, sample size, study duration, and the dose of flaxseed or its derivatives used. Previous meta-analyses^47-49^ in this field have combined studies involving both healthy and non-healthy individuals. Baseline BP levels can differ significantly between healthy individuals and those suffering from metabolic disorders, thereby influencing the results of these investigations. Additionally, no research has specifically focused on the effects of flaxseeds on individuals with risk factors for heart disease. In present analysis, only studies employing a parallel design were considered, as variations in study design may significantly affect the outcomes.

 This systematic review and meta-analysis aim to examine the scientific evidence regarding the effects of flaxseed supplementation on BP in patients with CVD risk factors.

## Methods

 We followed the Preferred Reporting Items for Systematic Reviews and Meta-Analyses (PRISMA) guidelines to conduct and report this paper.^50^

###  Literature Search

 The following global scientific databases were explored for pertinent articles published until April 2024: Cochrane Central Library, Web of Science, Scopus, and PubMed. The subsequent keywords were utilized in the search: (flax OR flaxseed OR linseed OR lignan OR *Linum usitatissimum*) AND (BP OR blood pressure OR SBP OR systolic blood pressure OR DBP OR diastolic blood pressure OR hypertension). In the search strategy, we included the aforementioned MeSH terms and keywords without language limitations. In addition, we searched through the references of the articles that were included and recent reviews to identify papers that fulfilled the eligibility requirements. The complete search strategies are presented in Supplementary file, Table S1.

###  Study selection

 Two researchers (R.HR. and I.A) conducted an independent examination of titles, abstracts, and subsequently, full texts to identify potentially qualifying articles. Any discrepancies were resolved through consensus with the third author (P.K). The following criteria were used to include eligible studies in this meta-analysis: (1) the studies should be randomized controlled trials (RCTs) with a parallel design; (2) they should investigate the effect of flaxseed on BP among individuals exhibiting risk factors for CVD such as dyslipidemia, T2DM, MetS, HTN, pre-diabetes, nonalcoholic fatty liver (NAFLD), or obesity; (3) they should provide baseline and end-trial BP measurements for both flaxseed and control groups; and (4) they must involve a flaxseed supplementation duration of no less than one week., Trials that did not provide adequate data regarding BP measurements for both intervention and control groups as well as non-clinical studies, and uncontrolled RCTs were excluded from present meta-analysis. Furthermore, studies examining the effects of flaxseed in conjunction with other interventions such as herbs or substances, as well as those involving children, adolescents, pregnant women, and lactating women, were also excluded.

###  Data extraction

 The data was meticulously extracted through the utilization of a standardized form that was specifically designed to ensure consistency and accuracy in the collection process. Two investigators (D.N and H.M.H) conducted an independent assessment of each manuscript that satisfied the established eligibility criteria and gathered the following pertinent information: the surname of the primary author, the year of publication, the geographical setting, mean age, body mass index (BMI), and gender of participants, duration of follow-up, overall sample size, dose, type of flaxseed and control group, the overall health condition of participants, as well as the mean and standard deviation (SD) of SBP and DBP at both the baseline and concluding phases of the research. Any discrepancies were resolved through the agreement reached by the third author (P.K).

###  Quality assessment

 The Cochrane Risk of Bias tool^51^ was employed to assess the methodological quality of the included RCTs. This instrument comprised seven distinct domains: random sequence generation, allocation concealment, blinding of participants and personnel, blinding of outcome assessment, incomplete outcome data, selective reporting, and other sources of bias. Each domain was categorized as exhibiting a low, high, or unclear risk of bias. Two independent reviewers (D.N and H.M.H) conducted a thorough evaluation of the assessment’s methodological quality, ensuring a rigorous and unbiased analysis, and any discrepancies identified in the Cochrane Risk of Bias ratings were addressed through discussion.

###  Evaluation of evidence strength

 The assurance of evidence was appraised employing the Grading of Recommendations Assessment, Development, and Evaluation (GRADE) framework. This assessment focused on several domains: risk of bias, inconsistency, indirectness, imprecision, and publication bias.^52^ The quality of the evidence was rated as high, moderate, low and very low.

###  Statistical analysis

 In the initial analysis, we assessed the intergroup variations in BP alterations between the flaxseed and control groups, subsequently aggregating these findings. The effect size was examined through the application of the weighted mean difference (WMD) alongside a 95% confidence interval (CI). The data were subjected to analysis utilizing a random-effects model, which facilitated the possibility that the true effect may differ across various studies. The statistical heterogeneity was evaluated using the *I*^2^ index. We considered a value greater than 50% as denoting substantial heterogeneity. We performed a subgroup analysis to evaluate the influence of the intervention duration and the participants’ health status on the resultant outcomes. A non-linear dose-response analysis was conducted to examine the relationship between overall effect size and flaxseed dosage (g/day). The sensitivity of the findings to individual studies was scrutinized by systematically omitting one publication at a time from the analysis. In order to assess the potential existence of publication bias, the Egger’s test was employed. The computer software “STATA, version 11.2 (Stata Corp., College Station, TX, USA)” was employed for the execution of the meta-analyses of the data, with all statistical tests being two-sided and a significance level established at 0.05, unless indicated otherwise.

## Results

###  Summary of included studies

**Figure 1:**
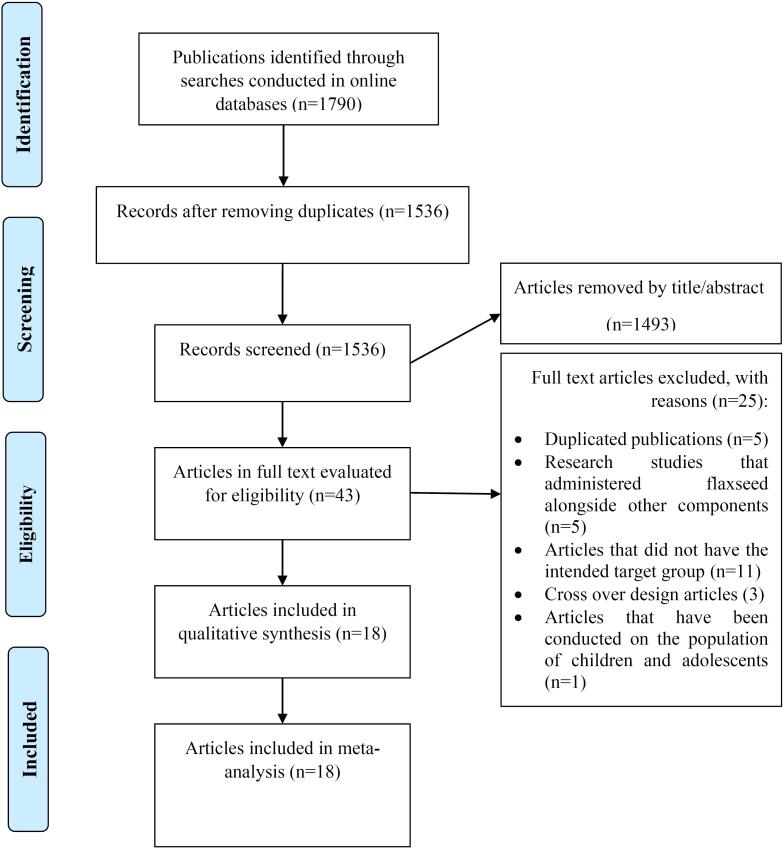
Flow diagram of the literature and screening process

 Out of the 1790 articles that were initially identified, only 43 full-text articles underwent a detailed evaluation. Nonetheless, 25 of these investigations were excluded for a variety of reasons (as illustrated in Figure 1). Consequently, only 18 articles,^29-46^ encompassing a total of 1298 subjects, fulfilled the inclusion criteria and were selected for subsequent qualitative and quantitative analysis.

###  Characteristics of the studies

Table 1 presents a comprehensive overview of the essential characteristics of the included papers. These articles were published between 2010 and 2022. A total of nine investigations were executed within the geographical confines of Iran, while three were undertaken in China. The remaining research engagements were carried out in India, Brazil, the United States of America, the Netherlands, and Cuba, respectively. The average age of the participants spanned from 22 to 66 years, and the baseline BMI exhibited variability from 23 to 32 kg/m^2^. One of the studies had male-only subjects while the remaining were with both genders. The time duration of included RCTs varied from 6 weeks to three months. Included studies were conducted in various populations, including subjects with coronary artery disease, T2DM, HTN, pre-diabetes, dyslipidemia, obesity, MetS, and NAFLD.

**Table 1:** Study characteristics of included studies

**First author** **(publication year, country)**	**Total participants (gender)**	**Mean age** **(year)**	**Mean BMI** **(kg/m**^2^**)**	**Duration** **(week)**	**Health condition**	**Intervention**	**Control **
Bhardwaj ^30^ (2022, India) a	76 (Male/Female)	57	25	24	Coronary artery disease	Blended flaxseed oil (30 g/d)	Sunflower oil
Bhardwaj ^30^ (2022, India) b	76 (Male/Female)	57.5	23	24	Hypertension	Blended flaxseed oil (30 g/d)	Sunflower oil
Toulabi ^42^ (2022, Iran)	76 (Male/Female)	52	29	12	Hypertension	Flaxseed (30 g/d)	Wheat flour
Yari ^45^ (2021, Iran) a	47 (Male/Female)	45	31	12	Metabolic syndrome	whole flaxseed (30 g/d) + lifestyle intervention	lifestyle intervention
Yari ^45^ (2021, Iran) b	44 (Male/Female)	45	31	12	Metabolic syndrome	whole flaxseed (30 g/d) + hesperidin (1 g/d) + lifestyle intervention	lifestyle intervention + hesperidin (1 g/d)
Rezaei ^39^ (2020, Iran)	68 (Male/Female)	43	30	12	NAFLD	Flaxseed oil (20 g/d)	Sunflower oil
Kuang ^37^ (2020, China)	51 (Male/Female)	22	26	8	Overweight and obese	Flaxseed meal (100g/d)	Control biscuits
Yang ^44^ (2019, China)	74 (Male/Female)	57	26	12	Hypertension	Flaxseed oil (2.5 g/d)	Corn oil
Pieters ^38^ (2019, Netherlands)	59 (Male/Female)	60	30	12	Overweight and obese	Refined cold-pressed flaxseed oil (1 g/d)	Sunflower oil
Saleh-Ghadimi ^41^ (2019, Iran)	40 (Male/Female)	55	30	10	Coronary artery disease	Flaxseed oil (5 g/d)	Milk
Hasaniani ^34^ (2019, Iran)	57 (Male/Female)	53	29	8	T2DM	Flaxseed (30 g/d)	Plain yogurt
Haghighatsiar ^33^ (2019, Iran)	80 (Male/Female)	43	28	8	Dyslipidemic and hypertensive patients	Flaxseed sachet (36g/d)	Placebo sachet
Akrami ^29^ (2017, Iran)	52 (Male/Female)	48	NR	7	Metabolic syndrome	Flaxseed oil (25 ml/d)	Sunflower oil
Yari ^46^ (2016, Iran)	44 (Male/Female)	45	30	12	Metabolic syndrome	Brown milled flaxseed (30g/d)	lifestyle advice
Javidi ^35^ (2016, Iran)	62 (Male/Female)	51	27	12	Prediabetic	Flaxseed powder (40 g/d)	Control
Cassani ^31^ (2015, Brazil)	27 (Male)	36	32	6	Cardiovascular risk factors	Flaxseed powder (60 g/d)	Raw rice powder
Katare ^36^ (2013, India)	50 (Male/Female)	52	28	12	Dyslipidemia	Roasted flaxseed powder (30 g/d)	Control
Rodriguez-Leyva ^40^ (2013, Cuba)	86 (Male/Female)	66	NR	24	Hypertension	Milled flaxseed (30g/d)	Wheat flour
Dewell ^32^ (2011, USA)	40 (Male/Female)	49	30	8	Metabolic syndrome	Flaxseed oil (6.6 g/d)	Soybean oil
Wu ^43^ (2010, China)	189 (Male/Female)	48	25	12	Metabolic syndrome	Flaxseed (30g/d) + lifestyle counseling	Lifestyle counseling

BMI, body mass index; T2DM, type 2 diabetes mellitus; NAFLD, nonalcoholic fatty liver; NR, not reported

###  Risk of bias, and grade assessment

 Most of the included publications were of high methodological quality, according to the Cochrane Risk of Bias checklist. Comprehensive information regarding the quality assessment is reported in Table 2. According to the GRADE approach both outcomes were rated at moderate levels of evidence. The level of evidence was downgraded due to serious imprecision limitations. More details of grade assessment are showed in Table 3.

**Table 2:** Literature quality assessment based on Cochrane guidelines

**First author** **(publication year)**	**Random Sequence Generation**	**Allocation concealment**	**Blinding of participants, personnel **	**Blinding of outcome assessment**	**Incomplete outcome data**	**Selective outcome reporting**	**Other sources of bias**
Bhardwaj ^30^ (2022)	L	L	L	H	L	L	H
Toulabi ^42^ (2022)	L	L	L	L	L	L	L
Yari ^45^ (2021)	L	U	L	L	U	U	L
Rezaei ^39^ (2020)	L	L	L	H	L	L	L
Kuang ^37^ (2020)	L	L	L	H	L	L	L
Yang ^44^ (2019)	L	L	L	L	L	L	L
Pieters ^38^ (2019)	L	L	L	H	L	L	L
Saleh-Ghadimi ^41^ (2019)	L	L	L	L	L	L	L
Hasaniani ^34^ (2019)	L	H	L	H	L	L	L
Haghighatsiar ^33^ (2019)	L	L	L	L	L	L	L
Akrami ^29^(2017)	L	L	L	H	U	L	U
Yari ^46^ (2016)	L	U	L	L	U	U	L
Javidi ^35^ (2016)	L	L	L	H	U	L	L
Cassani ^31^ (2015)	L	L	L	H	H	U	L
Katare ^36^ (2013)	U	U	L	H	U	U	U
Rodriguez-Leyva ^40^ (2013)	L	L	L	H	L	L	U
Dewell ^32^ (2011)	L	U	L	H	U	U	L
Wu ^43^ (2010)	L	L	L	U	L	U	U

U, unclear risk of bias; L, low risk of bias; H, high risk of bias

**Table 3:** Quality of the evidence evaluated by GRADE

**Outcome**	**Certainty assessment**					**Certainty**
	**Risk of bias**	**Inconsistency**	**Indirectness**	**Imprecision**	**Publication bias**	
SBP	No serious limitation	No serious limitation	No serious limitation	Serious limitation	No serious limitation	⨁⨁◯⨁ Moderate
DBP	No serious limitation	No serious limitation	No serious limitation	Serious limitation	No serious limitation	⨁⨁◯⨁ Moderate

SBP, systolic blood pressure; DBP, diastolic blood pressure

###  Effect of flaxseed supplementation on systolic blood pressure

**Figure 2:**
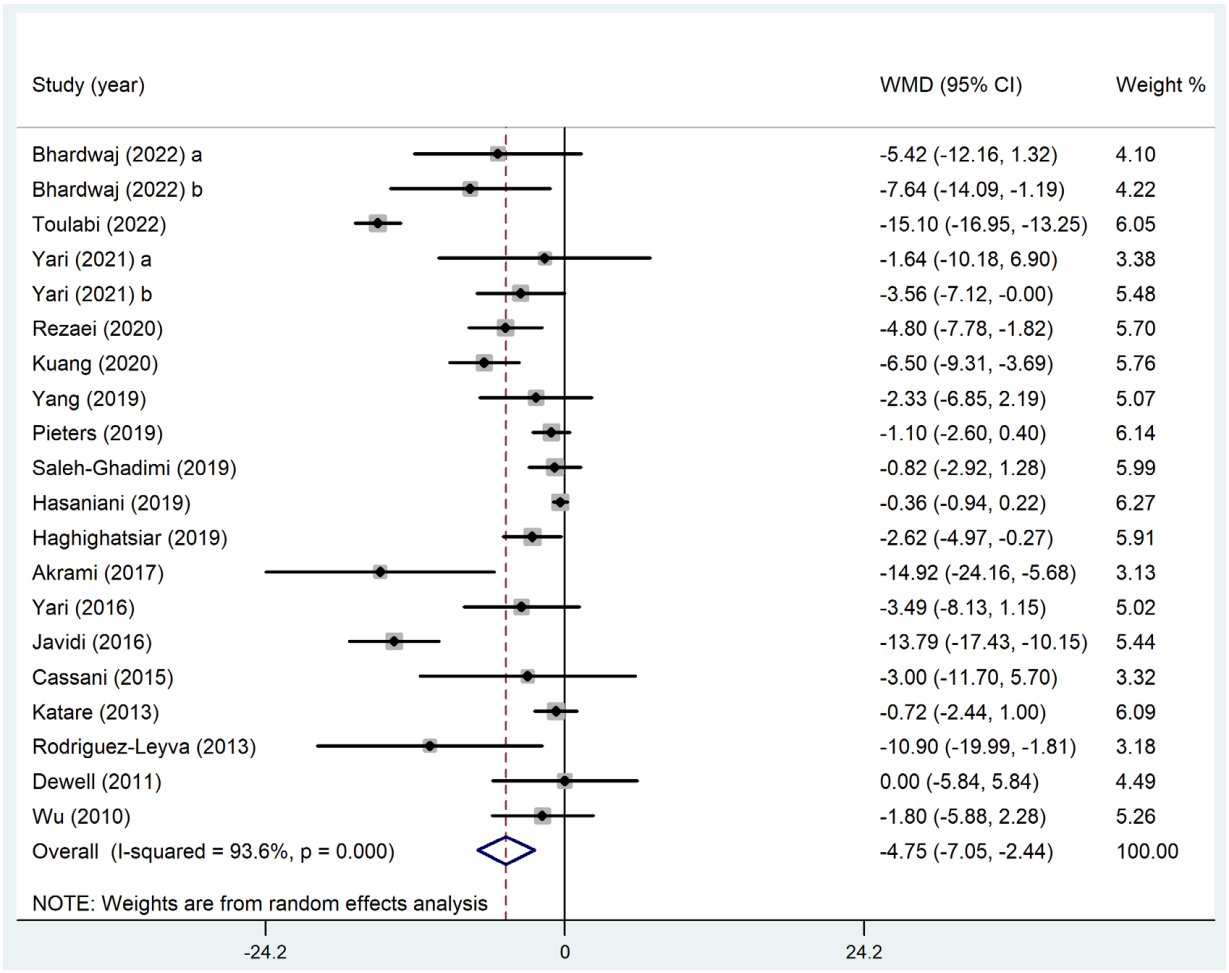
Forest plot illustrating the impact of flaxseed supplementation on systolic blood pressure in participants with cardiovascular disease risk factors

 A comprehensive analysis of 18 trials which included 20 arms, suggested significant benefits to flaxseed supplementation regarding systolic BP (SBP) levels (WMD: -4.75 mmHg, 95% CI: -7.05 to -2.44, *P* ≤ 0.001). However, the analysis indicated significant heterogeneity between these 18 trials (*I*^2^ = 93.6%, *P* < 0.001) (Figure 2). When studies were evaluated based on the study duration or the health status of the participants, a significant effect was recorded on SBP in all subgroups except in subjects who had dyslipidemia (WMD: -1.49 mmHg, 95% CI: -3.32 to 0.34, *P* = 0.1). Furthermore, heterogeneity between studies in subsets of participants with MetS and dyslipidemia decreased significantly (*I*^2^ = 37.2%, *P* = 0.15 and *I*^2^ = 38.9%, *P* = 0.20; respectively) (Table 4).

**Table 4:** Result of subgroup analysis of included studies in meta-analysis.

**Sub-grouped by**	**No. of arms**	**Effect size**^1^	**95% CI**	**P for effect size**	**I**^2^** (%)**	**P for heterogeneity**
**SBP**						
Health status						
Dyslipidemia	2	-1.49	(-3.32 to 0.34)	0.1	38.9%	0.20
Hypertension	4	-9.06	(-16.41 to -1.70)	0.01	89.7%	˂0.001
Metabolic syndrome	6	-3.29	(-6.03 to -0.55)	0.01	37.2%	0.15
Other	8	-4.20	(-6.76 to -1.63)	0.001	90.7%	˂0.001
Intervention duration						
> 10 weeks	13	-5.51	(-9.06 to -1.96)	0.002	93.8%	˂0.001
≤ 10 weeks	7	-2.80	(-5.11 to -0.49)	0.01	79.8%	˂0.001
**DBP**						
Health status						
Dyslipidemia	2	-0.84	(-1.61 to -0.07)	0.03	0.0%	0.76
Hypertension	4	-7.05	(-11.28 to -2.83)	0.001	83.7%	˂0.001
Metabolic syndrome	6	-1.24	(-2.42 to -0.05)	0.04	0.0%	0.82
Other	8	-3.27	(-5.34 to -1.21)	0.002	95.1%	˂0.001
Intervention duration						
> 10 weeks	13	-3.81	(-5.51 to -2.11)	˂0.001	89.7%	˂0.001
≤ 10 weeks	7	-1.65	(-4.01 to 0.71)	0.17	92.2%	˂0.001

^1^Calculated by Random-effects model

###  Effect of flaxseed supplementation on diastolic blood pressure

**Figure 3:**
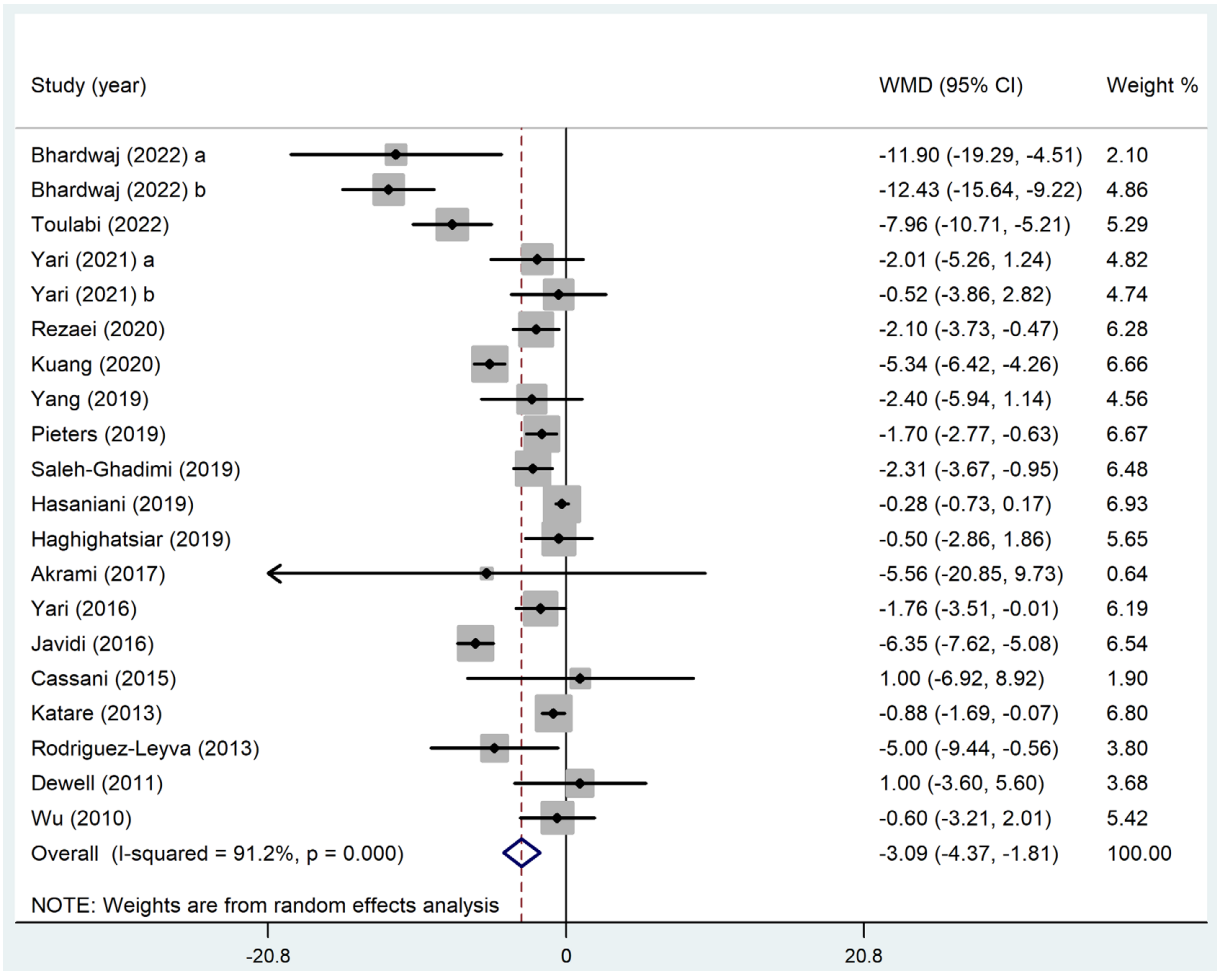
Forest plot illustrating the impact of flaxseed supplementation diastolic blood pressure in participants with cardiovascular disease risk factors

 A total of 18 studies (20 arms) reported diastolic BP (DBP) outcomes. The overall WMD analyzed through a random-effects model indicated a statistically significant improvement in DBP among subjects receiving flaxseed compared to the control group (WMD: -3.09 mmHg, 95% CI: -4.37 to -1.81, *P* ≤ 0.001). Nevertheless, it is important to note that there existed significant heterogeneity among the RCTs (*I*^2^ = 91.2%, *P* < 0.001)(Figure 3). In order to find out the source of this heterogeneity, sub-group analysis was done. The findings indicated that flaxseed was effective in lowering DBP in all subgroups, except for interventions lasting less than 10 weeks (WMD: -1.65 mmHg, 95% CI: -4.01 to 0.71, *P* = 0.17). In addition, the heterogeneity among studies in the subgroup of subjects with MetS and dyslipidemia dropped immensely (*I*^2^ = 0.0%, *P* = 0.82 and *I*^2^ = 0.0%, *P* = 0.76, respectively)(Table 4).

###  Dose-response analyses 

**Figure 4:**
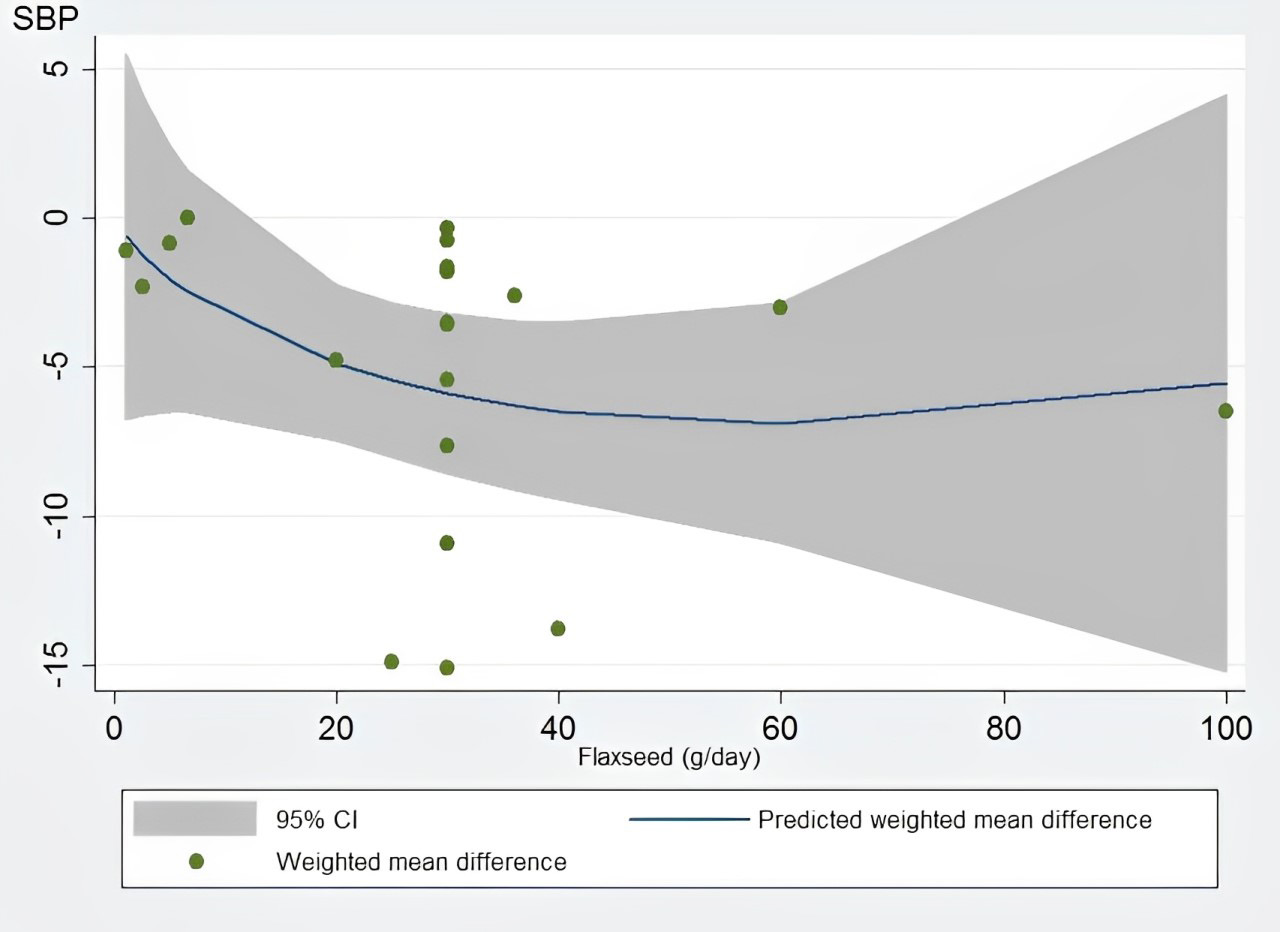
Non-linear dose-responses between flaxseed and weighted mean difference in systolic blood pressure (mmHg). The 95% confidence interval (CI) is depicted in the shaded regions.

**Figure 5:**
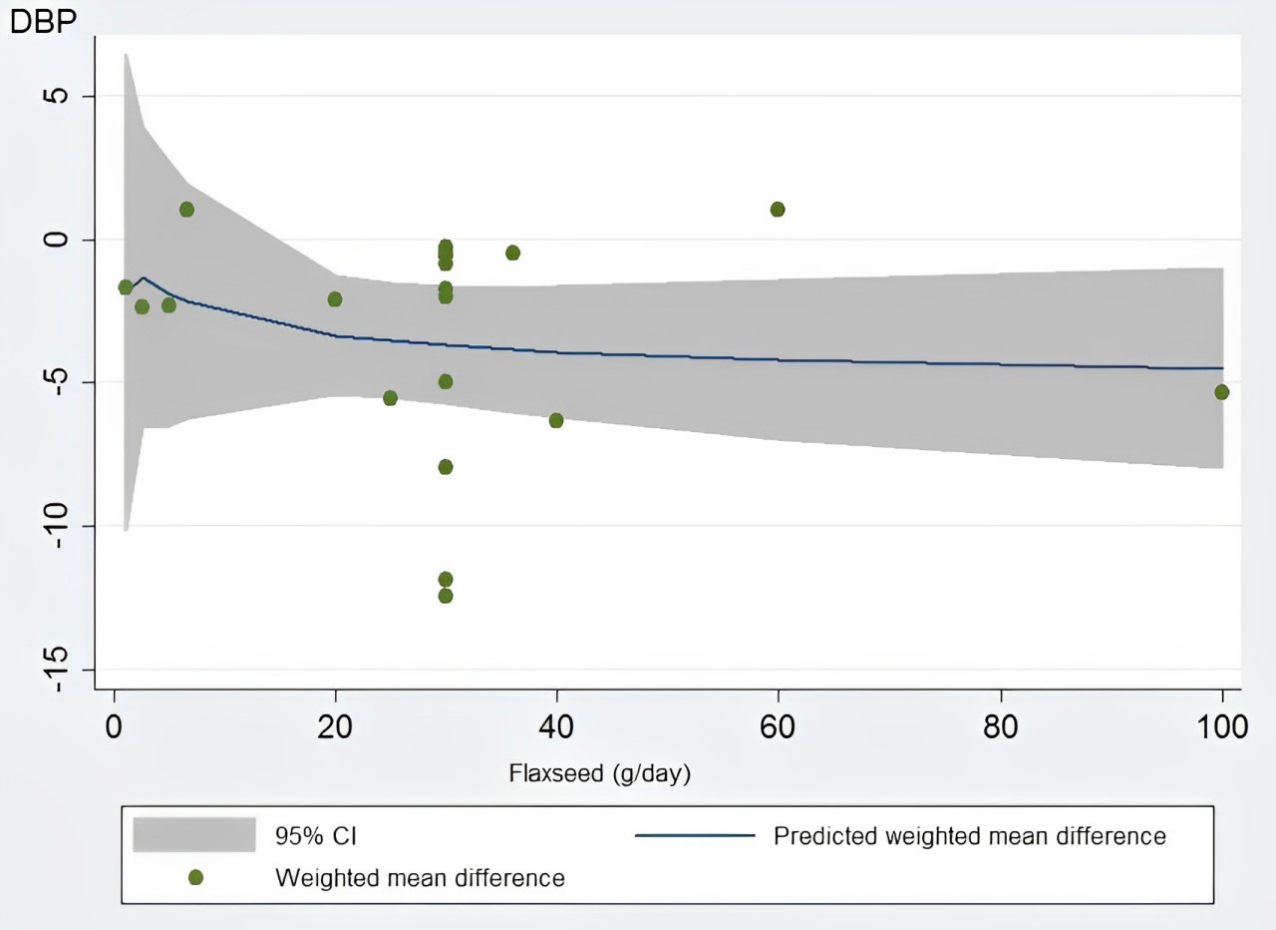
Non-linear dose-responses between flaxseed and weighted mean difference in diastolic blood pressure (mmHg). The 95% confidence interval (CI) is depicted in the shaded regions

 Dose-response analysis indicated that flaxseed supplementation did not significantly affect SBP (*P* = 0.15) and DBP (*P* = 0.64) levels based on the dosage. It can be concluded from the dose-response analysis that the optimum level of flaxseed that leads to the greatest observed changes in SBP and DBP was 30 grams daily (Figures 4 and 5).

###  Sensitivity analysis and publication bias

 After systematically removing each study, it was found that the overall effects of flaxseed supplementation on both outcomes were not significantly altered. Egger’s regression asymmetry test for the assessment of the publication bias was performed in the present meta-analysis. The results showed no evidence of publication bias in the studies that examined the effect of flaxseed supplementation on SBP (*P* = 0.05) and DBP (*P* = 0.05).

## Discussion

 This meta-analysis included 18 RCTs that assessed the effect of flaxseed supplementation on BP in patients with CVD risk factors. The combined effects of these trials showed that there were significant differences in SBP and DBP reduction with flaxseed supplementation as compared to the control group. But, it must be mentioned that the heterogeneity across the studies included was very high and by subgrouping only in some subsets the heterogeneity was decreased. For further studies, it should be noted that the interpretation of subgroups should be interpreted with care due to the low power of the subgroup analyses.

 The results of this meta-analysis are consistent with three previous meta-analyses^47-49^ that investigated the BP-lowering effects of flaxseed in adults. However, unlike those studies, the present analysis excluded healthy subjects. On the other hand, a different meta-analysis^53^ focused specifically on patients with MetS and related disorders and found that flaxseed can lower SBP, but does not affect DBP. The discrepancies between the results of different meta-analyses on the anti-hypertensive properties of flaxseed may stem from differences in the study participants. It stands to reason that flaxseed may not produce the same effects in individuals who are healthy as in those who have health issues. Additionally, individuals with HTN may experience more significant reductions in their BP levels as a result of consuming flaxseed.

 The underlying mechanisms responsible for the reduction of BP attributed to flaxseed remain inadequately elucidated. It is hypothesized that multiple factors may play a role in this reduction, encompassing a cardiac depressant action, a diuretic effect, and the presence of calcium channel-blocking attributes.^40,54,55^ Flaxseed is abundant in ligands, dietary fibers, and phytoestrogens, all of which have demonstrated efficacy in the modulation of BP through a variety of mechanisms.^47^ The alpha-linolenic acid (ALA) found in flaxseeds has the capacity to induce vasodilation, consequently resulting in a decrease in BP.^56^ Flaxseed possesses the remarkable ability to lower BP through a multifaceted mechanism that involves the modulation of blood lipid profiles, the augmentation of insulin resistance, as well as the promotion of beneficial gut microflora, all of which can be attributed to the presence of its soluble fiber components.^53,57^ Furthermore, flaxseed meal protein contains peptide bonds rich in arginine, which can help in generating nitric oxide and other biochemical reactions to improve BP levels.^48,58^

 The results indicate that flaxseed supplementation has a positive effect on BP in patients with CVD risk factors. This improvement in BP may help alleviate the clinical and public health issues associated with HTN and its related complications. Stamler et al^59^ demonstrated that even a reduction of 2 to 3 mmHg in SBP can result in a 4% decrease in CVD mortality. Additionally, Ogihara et al^60^ reported that such a reduction in SBP can lead to a 6.4% decrease in mortality from cerebrovascular disease. Therefore, based on the average effect size observed in this study, it can be concluded that the BP-lowering effects of flaxseed are both clinically significant and highly effective. Eventually, flaxseed can be an auxiliary option for people with cardiovascular risk factors, in conjunction with their diet and BP medications.

 Flaxseed is predominantly regarded as safe for ingestion, as research conducted on both animal models and human subjects has not indicated the occurrence of significant adverse effects. Nonetheless, excessive consumption of flaxseed may lead to detrimental outcomes such as diarrhea and hypersensitivity reactions. People with coagulation disorders, as well as pregnant or breastfeeding women and children, should consume flaxseed and its derivatives with caution.^53,61^

 Given the identified limitations, it is crucial to evaluate the findings from this comprehensive meta-analysis with caution. Initially, the study groups had a diverse composition, meaning that the research included subjects with various demographic and clinical characteristics, such as differing ages, genders, types of interventions, and initial health statuses. It is of particular significance to acknowledge this in relation to BP levels, given that certain participants in the study exhibited normotensive characteristics while others presented with hypertensive conditions. Secondly, BP was assessed as a secondary outcome in the majority of studies, which may have exerted an influence on the results. Thirdly, the disparate effects of flaxseed on male and female subjects remain undetermined, as the majority of studies included both genders of participants together. Ultimately, the majority of trials failed to report or analyze confounding factors and their corresponding impacts, thereby inhibiting their evaluation in our study. Due to processing delays for submissions pertaining to studies conducted outside the UK, we did not formally register the protocol for the present study in the PROSPERO registry. This lack of registration may potentially introduce a source of bias within the scope of this review. Notwithstanding this limitation, we meticulously formulated and conducted this review and meta-analysis in accordance with Cochrane guidelines.

## Conclusion

 This comprehensive meta-analysis of RCTs revealed substantial decreases in both SBP and DBP as a result of flaxseed supplementation. Given the significant heterogeneity, it is crucial to interpret the current results with careful consideration. In addition, the mechanisms behind this effect remain to be elucidated. Consequently, it is pertinent to undertake studies with a long duration, particularly among individuals diagnosed with HTN, to ascertain the potential for any interactions, whether advantageous or adverse, with the standard pharmacological treatment protocols for HTN.

## Competing Interests

 The authors assert the absence of any potential conflicts of interest.

## Ethical Approval

 The investigation constitutes a comprehensive systematic review and meta-analysis; therefore, ethical approval was not deemed necessary.

## Supplementary Files


Table S1. Search terms
