# Supplementary material for: Efficacy of flaxseed in reducing blood pressure among patients with cardiovascular risk factors: A systematic review and meta-analysis of parallel randomized controlled trials
Source: J Cardiovasc Thorac Res. 2025 Mar 18;17(1):1–11. doi: 10.34172/jcvtr.025.33280 (PMC12068798; doi:10.34172/jcvtr.025.33280)
Supplement: Supplementary file 1 — Table S1. Search terms [file jcvtr-17-1-s001.pdf]

J Cardiovasc Thorac Res. 2025;17(1):1-S1
doi: 10.34172/jcvtr.025.33280
http://jcvtr.tbzmed.ac.ir

Table S1. Search terms

**PubMed**
("flax" OR "flaxseed" OR "linseed" OR "lignan" OR "*Linum usitatissimum*")<br>AND<br>("blood pressure" OR "systolic blood pressure" OR "diastolic blood pressure" OR "hypertension" OR "cardiovascular disease")
**Scopus**
("flax" OR "flaxseed" OR "linseed" OR "lignan" OR "*Linum usitatissimum*")<br>AND<br>("blood pressure" OR "systolic blood pressure" OR "diastolic blood pressure" OR "hypertension" OR "cardiovascular disease"):ti,ab,kw
**Web of Science**
#1. TS = (flax OR flaxseed OR linseed OR lignan OR *Linum usitatissimum*)<br>#2. TS = (blood pressure OR systolic blood pressure OR diastolic blood pressure OR hypertension OR cardiovascular disease)<br>#3. #1 AND #2
**Cochrane Library**
#1 flax OR flaxseed OR linseed OR lignan OR *Linum usitatissimum*: ti,ab,kw<br>#2 blood pressure OR systolic blood pressure OR diastolic blood pressure OR hypertension OR cardiovascular disease): ti,ab,kw
